# Electrophysiological Correlates of Morphological Neuroplasticity in Human Callosal Dysgenesis

**DOI:** 10.1371/journal.pone.0152668

**Published:** 2016-04-07

**Authors:** Vladimir V. Lazarev, Myriam de Carvalho Monteiro, Rodrigo Vianna-Barbosa, Leonardo C. deAzevedo, Roberto Lent, Fernanda Tovar-Moll

**Affiliations:** 1 National Institute of Women, Children and Adolescents Health Fernandes Figueira, Oswaldo Cruz Foundation, Rio de Janeiro, Brazil; 2 Institute for Biomedical Sciences, Federal University of Rio de Janeiro, Rio de Janeiro, Brazil; 3 D’Or Institute for Research and Education (IDOR), Rio de Janeiro, Brazil; University of Montreal, CANADA

## Abstract

In search for the functional counterpart of the alternative Probst and sigmoid bundles, considered as morphological evidence of neuroplasticity in callosal dysgenesis, electroencephalographic (EEG) coherence analysis was combined with high resolution and diffusion tensor magnetic resonance imaging. Data of two patients with callosal agenesis, plus two with typical partial dysgenesis with a remnant genu, and one atypical patient with a substantially reduced genu were compared to those of fifteen neurotypic controls. The interhemispheric EEG coherence between homologous nontemporal brain regions corresponded to absence or partial presence of callosal connections. A generalized coherence reduction was observed in complete acallosal patients, as well as coherence preservation in the anterior areas of the two patients with a remnant genu. jThe sigmoid bundles found in three patients with partial dysgenesis correlated with augmented EEG coherence between anterior regions of one hemisphere and posterior regions of the other. These heterologous (crossed) interhemispheric connections were asymmetric in both imaging and EEG patterns, with predominance of the right-anterior-to-left-posterior connections over the mirror ones. The Probst bundles correlated with higher intrahemispheric long-distance coherence in all patients. The significant correlations observed for the delta, theta and alpha bands indicate that these alternative pathways are functional, although the neuropsychological nature of this function is still unknown.

## Introduction

Callosal dysgenesis (CD) is characterized by a developmental defect of the corpus callosum [1,2], which may occur isolated or associated with other malformations [3], and may be total or partial [4]. Patients with CD can be asymptomatic or display different neuropsychological impairments [5]. Interestingly, however, the classical split-brain syndrome resulting from the surgical section of corpus callosum in adults [6] is not fully present in CD patients [7], who can perform many tasks requiring a considerable degree of interhemispheric communication [8, 9].

Anatomical evidence of white matter circuit reorganization in CD has been reported by many authors [2, 4, 10–12]. The main conclusion was that an early derangement of callosal development hinders axonal crossing and causes rewiring, providing the brain with a set of anomalous intra- and inter-hemispheric connections.

The functional brain repertoire in CD was previously explored by electroencephalographic (EEG) methods [13–16] and, more recently, by resting state functional magnetic resonance imaging (MRI) [17, 18]. Some of these previous studies have shown a decrease in interhemispheric connectivity in CD compared to controls [13–16, 18], while preserved bilateral functional networks in CD have also been reported [11, 17]. However, although advanced MRI techniques have underlined morphological features of the abnormal structural connectivity in CD [2, 4, 11], even prenatally [19], their functional correlates were much less systematically investigated [11]. Specifically, the functional correlates for the abnormal tracts connecting heterotopic cortical regions, such as the Probst (PB) and sigmoid (SB) bundles, remain unexplored.

In search for the functional counterpart of these bundles in CD, we applied EEG coherence analysis combined with high resolution anatomical MRI and diffusion tensor imaging (DTI) in patients, as compared with neurotypic controls. EEG coherence can quantitatively estimate the similarity of bioelectrical oscillations among brain areas, which indicates a high probability of functional interactions [20–24], especially in cases of callosal malformation [13–16]. Our objective was specifically to test the putative functional activity of the aberrant PB and SB present in patients with CD. The results have shown higher long-distance EEG coherence topographically corresponding to the PB and SB detected by MRI, including predominance of the same right-anterior-to-left-posterior connections over the mirror ones for the latter.

## Materials and Methods

### Participants

Five patients with CD (3 females; mean age of 15.26±9.5 years) and fifteen neurotypic control individuals (10 females; mean age of 11.0±5.3 years) were enrolled in the study, which was approved by the Ethics Committees of the National Institute of Women, Children and Adolescents Health Fernandes Figueira and of the D’Or Institute for Research and Education. All the volunteers and/or primary caregivers gave written informed consent for participation in the study.

### Neuroimaging

Acquisitions were conducted on an Achieva 3T Philips MR scanner (The Netherlands). An 8-channel SENSE head coil was used. Imaging protocol was composed by DTI and anatomical sequences, including a high resolution T1-weighed volumetric sequence (TR/TE = 7.2/3.4 s; voxel size = 1 mm^3^; field-of-view = 240 mm; 170 sagittal slices). For each subject, two diffusion-weighed images were acquired and averaged, using a single-shot, spin-echo, echo-planar sequence (TR/TE = 9,500/60 ms; voxel size = 2 mm^3^; field-of-view = 232 mm; 60 axial slices; scan time = 12 min), with diffusion sensitization gradients applied in 32 non-collinear directions (b factor = 1000sec/mm^2^).

Data processing was performed using DtiStudio [25] and PRIDE software (Philips Research Integrated Development Environment software, PRIDE research platform).

#### Diffusion tensor tractography (fiber-tracking)

Fiber-tracking was performed in DTIStudio and PRIDE software using the Fiber Assignment by Continuous Tracking (FACT) method [26]. The diffusion tensor for each voxel was calculated based on the eigenvectors (v1, v2, v3) and eigenvalues (λ1, λ2, λ3) using multivariate fitting and diagonalization [27]. Tracking was initiated at an FA value of 0.2 and was terminated when FA fell below 0.2 or the angle between two adjacent eigenvectors was greater than 40 degrees. In patients, tractography was performed in order to investigate the fiber topography of the PB and SB, using a multiple region-of-interest (ROI) approach [2, 11]. ROIs were placed in T1 images and loaded to the FA maps.

### Electroencephalography

The EEG was recorded (Bio-logic, USA) at 16 scalp points according to the International 10/20 System, with unilateral references to the corresponding earlobes and simultaneous registration of electrooculogram. The sampling frequency was 256 Hz. During EEG recording of 3–5 min duration, subjects were awake and resting, with their eyes closed. One-two min EEG fragments without artifacts were submitted to spectral analysis and calculation of coherence with EEG epochs of 1s duration (Fourier transform) (Brainsys-Neurometrics, Russia). Auto- and cross-power spectrum estimates were obtained by the averaging of sample spectra of disjoint EEG segments. The complex coherence function was calculated as a normalized cross-power spectrum [28]. Coherent connections were assessed by the coefficient of coherence (CCoh)—the square root of the computed magnitude squared coherence for the 5 frequency bands: delta—2 to 3.5 Hz, theta—3.5 to 7.5 Hz, alpha—7.5 to 13 Hz, beta1–13 to 20 Hz, and beta2–20 to 30 Hz. The CCoh was interpreted as an analogue of the frequency indexed correlation coefficient [29]. The threshold of independence between two signals was calculated. For fragments of 60 1-s disjoint EEG epochs, the magnitude squared coherence > 0.05, and respectively CCoh > 0.22, are significantly different from zero at the significance level of 0.05 [30, 31].

The 25 highest (H) CCohs in each subject were considered in order to circumvent a considerable inter-individual variability in the general level of EEG coherence and emphasize the individual coherence topography [23]. The minimum (threshold) value among these 25 HCCohs was considered as characteristic of an individual’s general level of coherence at a given frequency band. It was utilized as the divisor for the *absolute* CCohs values in order to ‘normalize’ them for more comparable and detailed presentation of each individual’s coherent connectivity by means of such *relative* CCohs [24].

Average CCohs were calculated for similar directions of coherence based on topographic orientations and selected anatomical and functional significance, in order to circumvent interindividual variability in EEG topography and relatively low spatial resolution of EEG. Averaging was performed for: (1) interhemispheric connections between homologous leads placed on various cerebral regions, (2) short-distance connections between adjacent leads of the same hemisphere, and (3) long-distance intrahemispheric connections topographically corresponding to the PB or the SB. In the latter case, we considered the heterologous (crossed) connections between anterior regions of one hemisphere (frontal and frontopolar leads) and posterior regions of the opposite hemisphere (occipital, parietal and central leads caudal to the SB crossing level).

Due to the small size of the patient group (since isolated CD is rare), we used a higher number of subjects in the control group to improve statistical reliability. In addition, we also report a descriptive analysis of each patient of our sample. The data were presented as the means ± standard deviations.

As for comparisons, since most of data in the groups did not show deviations in normality (Shapiro-Wilk test) and scedasticity (Levene’s test), parametric tests were applied in statistical analyses due to their higher robustness [32]. For each parameter, the statistical significance of the differences (p < 0.05) between three groups was calculated by one-factor ANOVA followed by the Tuckey’s post-hoc test when possible, using the general linear model paradigm. In the comparison between the two groups, unpaired T-test was used according to homogeneity of variances as estimated by the Levene’s test. The statistical analyses were performed by the SPSS package (version 15).

For the sake of simplicity and clarity, the connections between scalp points corresponding to PB and SB, with CCohs significantly different in patients as compared with controls were plotted in the illustrative brain maps, as only the difference in pairs of connections was of interest.

## Results

### Neuroimaging

**Fig 1:**
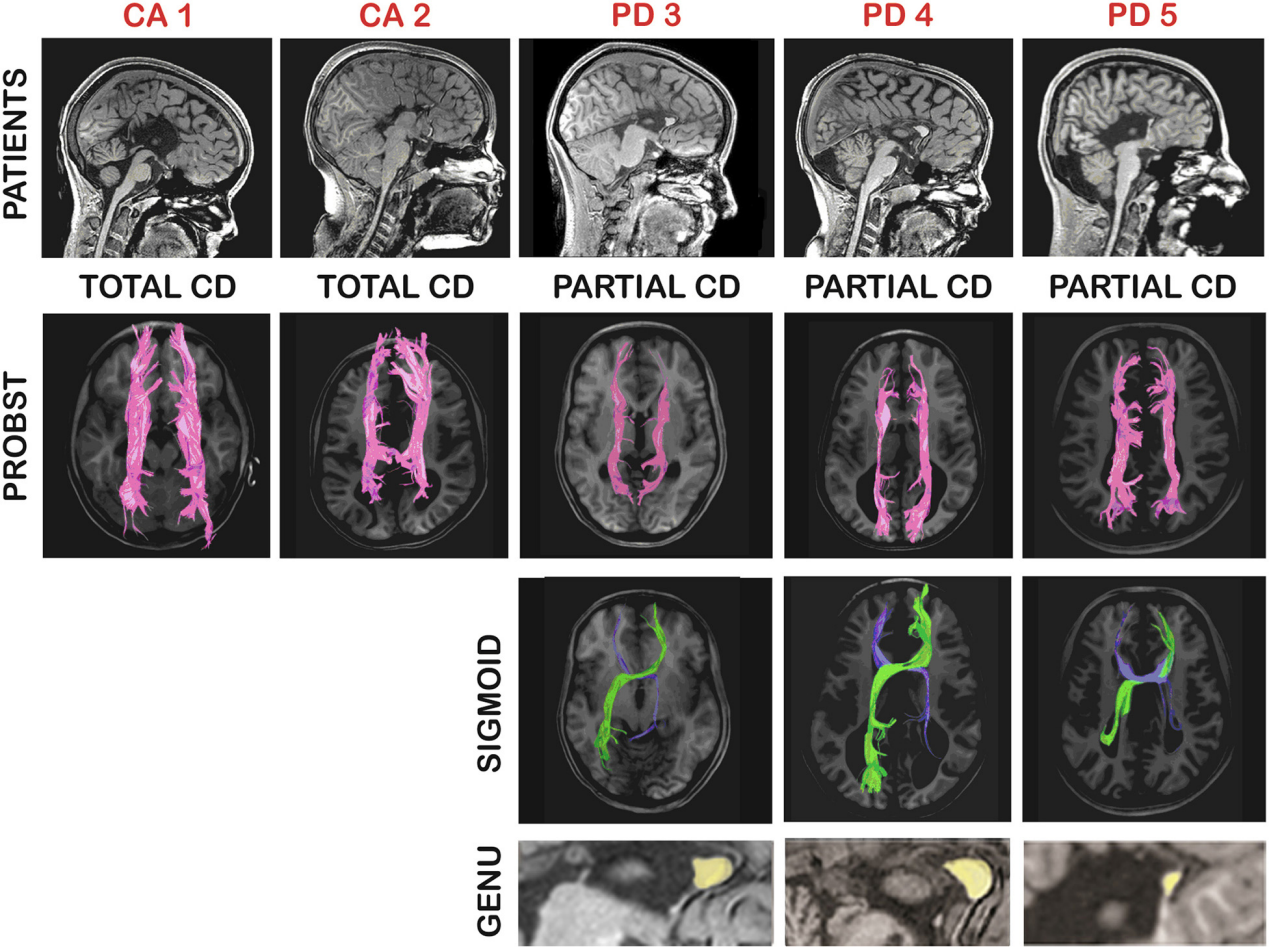
Summary of patients’ morphological characteristics. Each patient is identified by red letters (CA = callosal agenesis; PD = partial dysgenesis). Upper row, T1 sagittal images of patients. Middle rows, DTI tractography of the Probst (in purple) and the sigmoid (in green and blue) bundles. Lower row, amplified sagittal view to show the callosal remnants (in yellow) at genual topography (notice smaller dimension in PD 5).

Conventional structural MRI showed callosal agenesis (CA: total absence of the corpus callosum) in two patients (CA1 and 2) and partial CD (PD) in three patients (PD 3–5) whose genu was present as a remnant. It is important to note that PD 3 and 4 had a very similar remnant genu, whereas in PD 5, a much smaller remnant was observed. The typical anatomical features of CD were also present, including parallel, enlarged lateral ventricles, downward displacement of the cingulate gyrus, and radial sulci at the medial brain surface. Detailed anatomical inspection and DTI tractography reconstruction revealed bilateral PBs in all patients. In addition, interhemispheric SBs were observed in the three PD patients, always asymmetrical, with the right-anterior-to-left-posterior segment more robust than the opposite. No anatomical abnormalities were found in the controls. Patients’ morphological characteristics can be seen in Fig 1.

### Electroencephalography

#### Interhemispheric coherent connections

The two CA patients had a reduced percentage of interhemispheric connections. Regarding HCCohs, 16 and 20% of them were in the delta, 12.5 and 12% in the theta, and 12 and 24% in the alpha bands, as compared with 32 ± 10,6%, 29,3 ± 8,3% and 24,5 ± 12,7% in the PD patients and 38.0 ± 6.9%, 38.7 ± 5.9% and 32.5 ± 9.3% in the control group.

**Fig 2:**
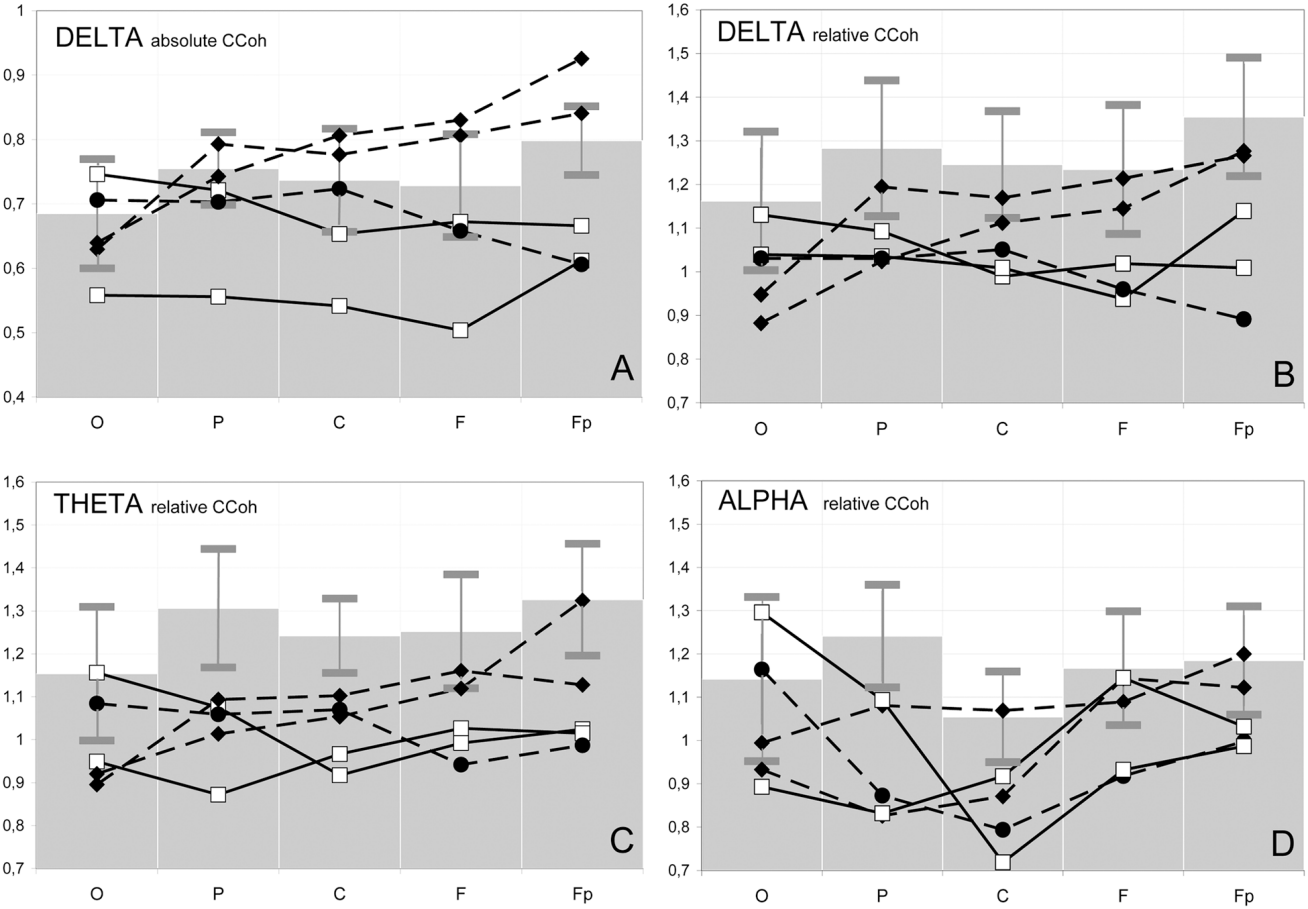
Electrophysiological correlates of interhemispheric communication. Coefficients of coherence (CCoh) between the EEG recorded from homologous leads on the hemispheres in the occipital (O), parietal (P), central (C), frontal (F) and frontopolar (Fp) areas in three frequency bands. *Absolute* values of coefficients are shown in **A**, and *relative* values in **B**, **C** and **D**. Solid lines: patients with callosal agenesis (CA 1 and 2 –white squares). Dashed lines: patients with partial callosal dysgenesis, including two with a typical, robust genu (PD 3 and 4—black rhombs) and one with a substantially reduced genu (PD5—black circles). Gray bars on the background represent mean ± standard deviation for CCoh values of the control subjects.

Concerning the coherence between homologous nontemporal areas, supposed to be the most dependent on the corpus callosum [33], only 57% of the CCohs in the CA patients pertained to the HCCohs in the delta, theta and alpha bands, against 68 and 94% in the PD and control groups, respectively (p<0.001 for the difference between each group of patients and controls). However, the absolute CCohs values were not sensitive enough to reflect peculiarities of the interhemispheric coherence in different brain regions. For example, in the delta band, they did not show any significant differences between the groups of all patients and controls (Fig 2A). The two CA patients (white squares), together with the atypical PD 5 patient with a substantially reduced genu (black circles) had CCohs (0.63 ± 0.03) significantly lower than the controls (0.80 ± 0.05; p<0.001; grey bars) only in the frontopolar region. In addition, when compared to the two typical PD patients (black rhombs), this group (two CA patients together with the atypical PD 5 patient) showed decreased CCohs in the frontopolar (0.88 ± 0.06 vs 0.63 ± 0.03; p<0.001) and frontal regions (0.82 ± 0.02 vs 0.61 ± 0.09; p = 0.03) as well.

No consistent differences in the above-mentioned characteristics were observed in the beta bands. The absolute CCohs between homologous temporal areas did not reach threshold level for HCCohs in both patients and controls.

The relative interhemispheric CCohs (that took into account each individual’s general level of coherence) were more consistent with the absence or partial presence of the corpus callosum (Fig 2). For example, the relative delta CCohs between homologous anterior areas in the typical PD patients 3 and 4 were below the controls’ averages (Fig 2B, black rhombs), in contrast to the larger absolute values (Fig 2A, black rhombs) probably due to increased general level of coherence (threshold CCohs 0.69 ± 0.04 against 0.60 ± 0.09 in the controls).

Along the same line, the group of two CA and one atypical PD 5 patients showed significantly reduced relative CCohs in all the homologous nontemporal pairs, except for the occipital ones, in the delta (1.01 ± 0.06 in patients vs. 1.28 ± 0.14 in controls, values averaged for the four regions; p≤ 0.05–0.01 for different regions) and theta (0.99 ± 0.06 vs. 1.28 ± 0.12; p≤ 0.01, respectively) bands, and in the parietal, central and frontopolar pairs in the alpha band (0.91 ± 0.09 vs. 1.16 ± 0.12, values averaged for the three regions; p<0.05–0.01) (Fig 2B, 2C and 2D, white squares and black circles). The relative CCohs of typical PD (PD3 and 4) were also significantly lower than in the control group only in the parietal and central regions for the theta band (1.07 ± 0.05 vs. 1.27 ± 0.11, values averaged for the two regions; p≤0.05) and in the parietal region for the alpha band (0.95 ± 0.18 vs. 1.24 ± 0.11; p = 0.02) while no difference with controls was detected in frontal and frontopolar regions (Fig 2C and 2D, white squares and black circles).

In general, the group of patients as a whole had significantly reduced relative CCohs for the delta, theta and alpha bands in the parietal and central areas (1.00 ± 0.03 vs. 1.23 ± 0.02 in controls, values averaged for the two regions and three bands; p≤0.04–0.001 for different bands in different regions) and no significant difference from the control group in the occipital region (1.02 ± 0.04 vs. 1.15 ± 0.02, respectively) (Fig 2B, 2C and 2D).

In the beta bands, no consistent differences between patients and controls were found.

#### EEG correlates of the callosal genu

In PD 3 and 4 (robust remnant genu present), the mean absolute CCohs between homologous frontal and frontopolar leads were substantially higher than between parietal and occipital ones (Fig 2, black rhombs). The anterior/posterior ratios of these CCohs were 1.21 ± 0.08 for delta and theta and 1.19 ± 0.13 for alpha bands, as compared with 1.06 ± 0.11, 1.05 ± 0.08 and 0.98 ± 0.12 in the controls, respectively (p = 0.02 for theta and p = 0.04 for alpha ratios). Controls’ anterior absolute delta CCohs were significantly lower than in patients (p = 0.05). In these PD patients, the anterior leads represented 67–80% of the HCCohs in all the above regions, against 52% in controls.

In the CA patients, the aforementioned anterior/posterior ratios were 0.96–1.03. The mean anterior delta and theta absolute (0.61 ± 0.08) and relative CCohs (1.02 ± 0.01) were significantly lower in patients than in controls (0.76 ± 0.06 and 1.29 ± 0.12, respectively; p<0.01; all values averaged for the two bands), with no difference for the posterior ones (Fig 2, white squares). The above percentage of the HCCohs in the anterior areas was 38–60%.

In atypical PD 5 (very small genual remnant), the delta and theta HCCohs between homologous points fell only on the parietal and occipital areas. The above anterior/posterior CCohs ratios were 0.84–0.94. The relative CCohs in the anterior regions (Fig 2B, 2C and 2D, black circles) were the lowest among all the individuals.

#### EEG correlates of sigmoid bundles

**Fig 3:**
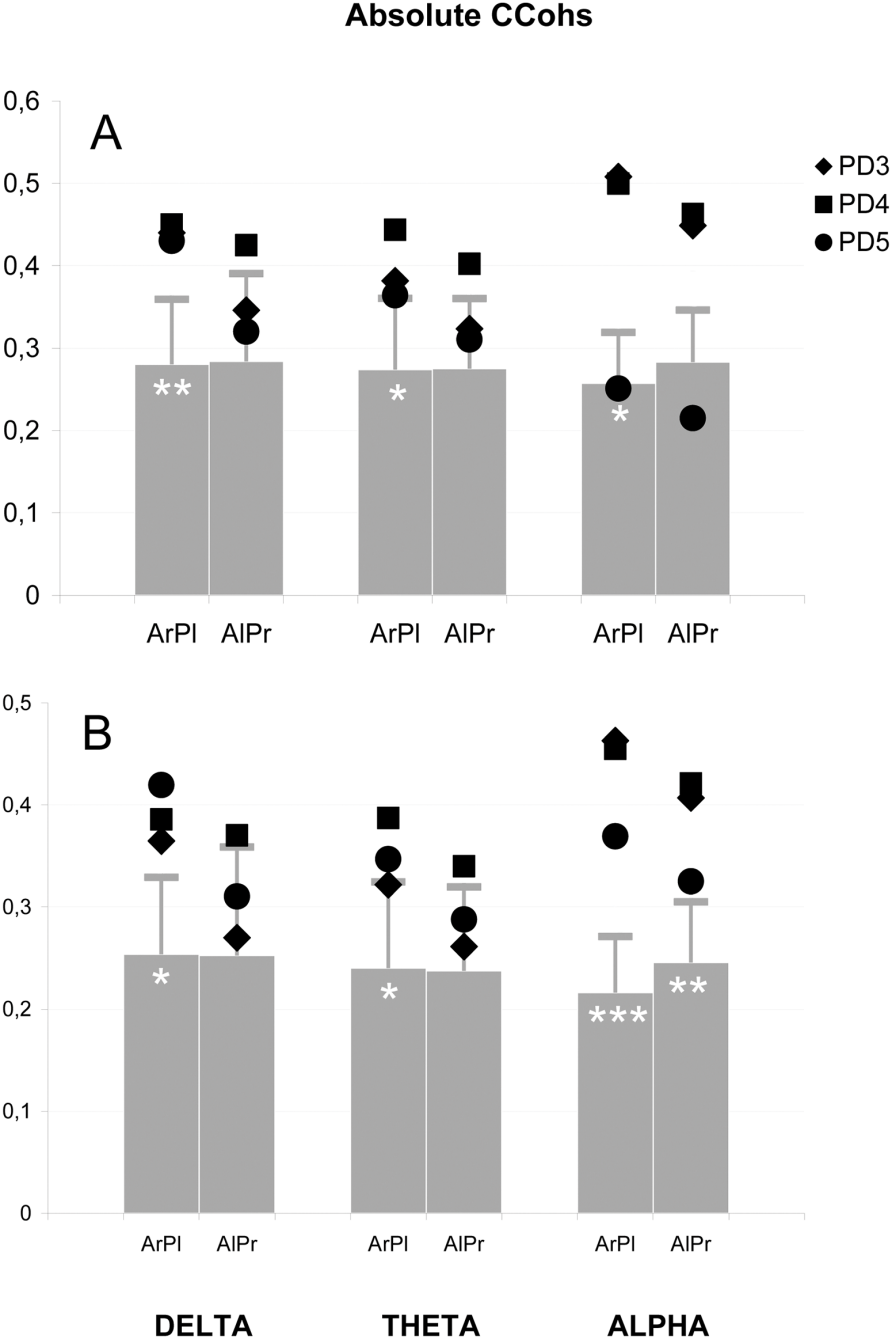
Electrophysiological correlates of the sigmoid bundles: Absolute coefficients of coherence (CCohs). Absolute values in three frequency bands averaged for: (**A**) coherent connections of anterior (A—frontopolar and frontal) with contralateral posterior (P—occipital, parietal and central) leads of the left (l) and right (r) hemispheres; (**B**) same connections excluding central leads. Grey bars represent the mean coefficients of the control group, horizontal lines depict standard deviations; black symbols indicate the coefficients of patients with robust genual remnant (PD 3 and 4) and residual remnant (PD 5); The number of white asterisks indicates the level of statistical significance of the difference between PD patients and control group: * p<0.05; ** p<0.01; *** p<0.001.

**Fig 4:**
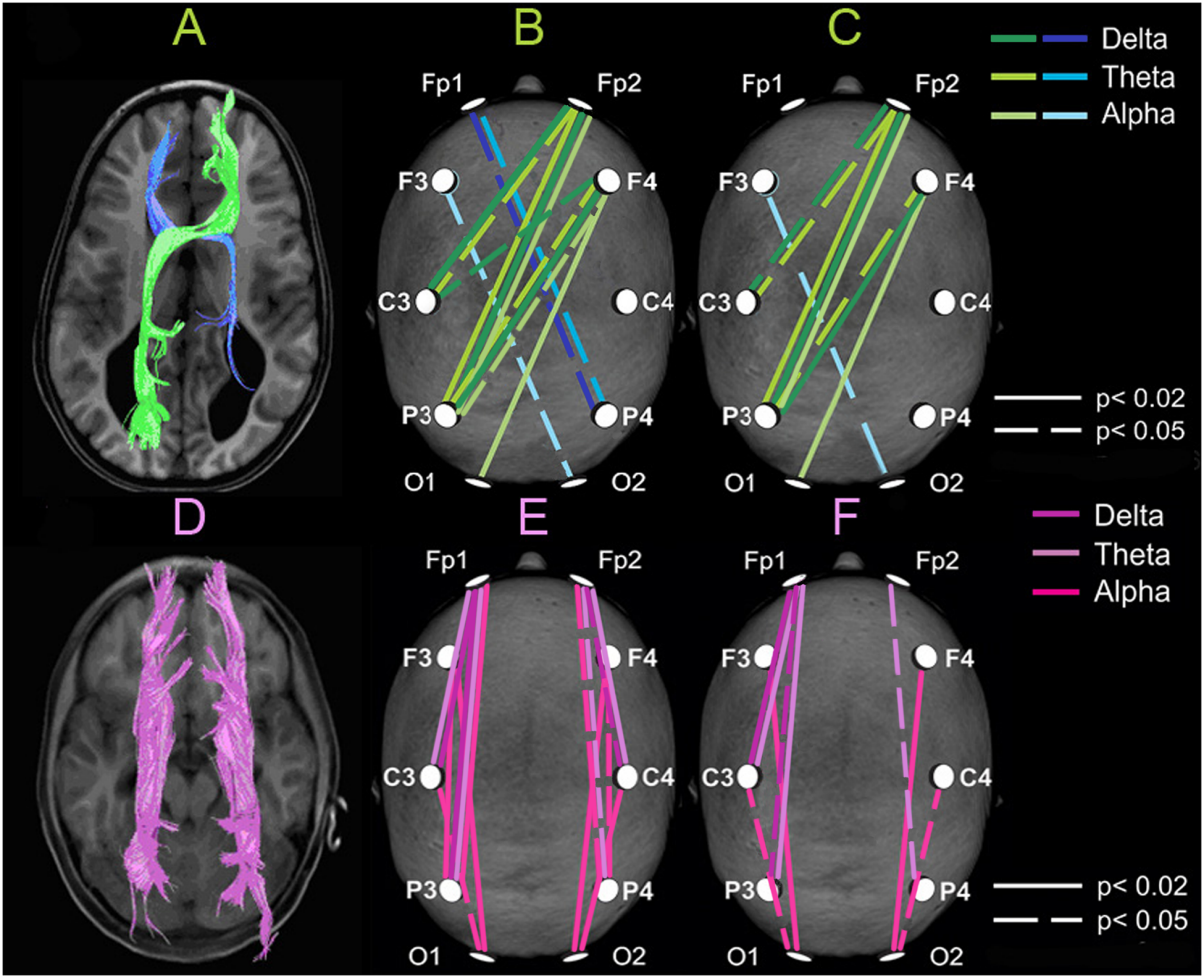
Correspondence of morphological features and electrophysiological correlates of the heterotopic aberrant bundles. **Upper row**. Tractography of sigmoid bundles (in green and blue) showing structural asymmetry of thickness (**A**). *Inter*hemispheric heterologous (crossed) coherent connections whose *absolute* (**B**) and *relative* (**C**) coefficients of coherence (see Fig 2) are significantly higher in 3 patients with partial callosal dysgenesis than in the control group, in 3 frequency bands. Notice the correspondence with morphology shown at left. **Lower row**. Tractography of Probst bundles (**D**, in purple). *Intra*hemispheric long-distance coherent connections whose *absolute* (**E**) and *relative* (**F**) coefficients of coherence are significantly higher in the group of five patients than in the control group, in 3 frequency bands. Left hemisphere is represented at left in all maps.

Fig 3A represents the *absolute* CCohs averaged for five coherent connections of the two anterior (frontopolar and frontal) leads of one hemisphere with the three posterior (occipital, parietal and central) ones of the other (except for the frontal-to-central crossed connections that are somewhat similar to adjacent homologous frontal and central pairs of leads), whose topographic orientations correspond to the SB (Figs 1, 4A and 4B). In the three PD patients with SB, the right-anterior-to-left-posterior mean CCohs in the delta (0.44 ± 0.004), theta (0.40 ± 0.04) and alpha (0.42 ± 0.14) bands were higher than in the control group (0.28 ± 0.08, 0.27 ± 0.09 and 0.26 ± 0.06; p = 0.01 for the delta and p = 0.02 for the alpha band, respectively). In the theta band, significant predominance of the PD patients over controls was observed only in comparison between these two groups (p = 0.03).

The mirror mean absolute CCohs were significantly augmented in the alpha band for the two PD patients with robust genual remnants (0.46 ± 0.01; p = 0.02) (Fig 3A). However, the PD patients as a whole showed such significant prevalence when the averaging was performed over the four long left-anterior-to-right-posterior connections excluding the right central lead (0.39 ± 0.05 vs. 0.25 ± 0.06; p = 0.004) (Fig 3B). In all the bands, the absolute CCohs for this topographic orientation in each PD patient was always lower than for the right-anterior-to-left-posterior one (Fig 3A and 3B) (p<0.02 in the theta and alpha bands for all three PD patients).

Among separate augmented absolute CCohs topographically corresponding to SB, the right-frontopolar-to-left-parietal connection was significant in the delta (0.48 ± 0.09 vs. 0.27 ± 0.11; p = 0.02), theta (0.46 ± 0.10 vs. 0.27 ± 011; p = 0.02) and alpha (0.43 ± 0.18 vs. 0.24 ± 0.09; p = 0.03) bands. Comparison of the PD patients with controls, excluding the group of CA patients without sigmoid bundles, showed various significantly augmented heterologous (crossed) CCohs such as the right-frontal-to-left-parietal ones in the delta (0.61 ± 0.05 in patients vs. 0.44 ± 011 in controls; p = 0.02), theta (0.59 ± 0.02 vs. 0.43 ± 0.12; p = 0.04) and alpha (0.52 ± 0.15 vs. 0.36 ± 0.10; p = 0.04) bands. The results of these comparisons are presented as illustrative maps in Fig 4 for all absolute (B) and relative (C) CCohs that were significantly higher in the PD patients than in the controls. These maps show augmented CCohs in both right-anterior-to-left-posterior and mirror axes, with lower number for the latter.

**Fig 5:**
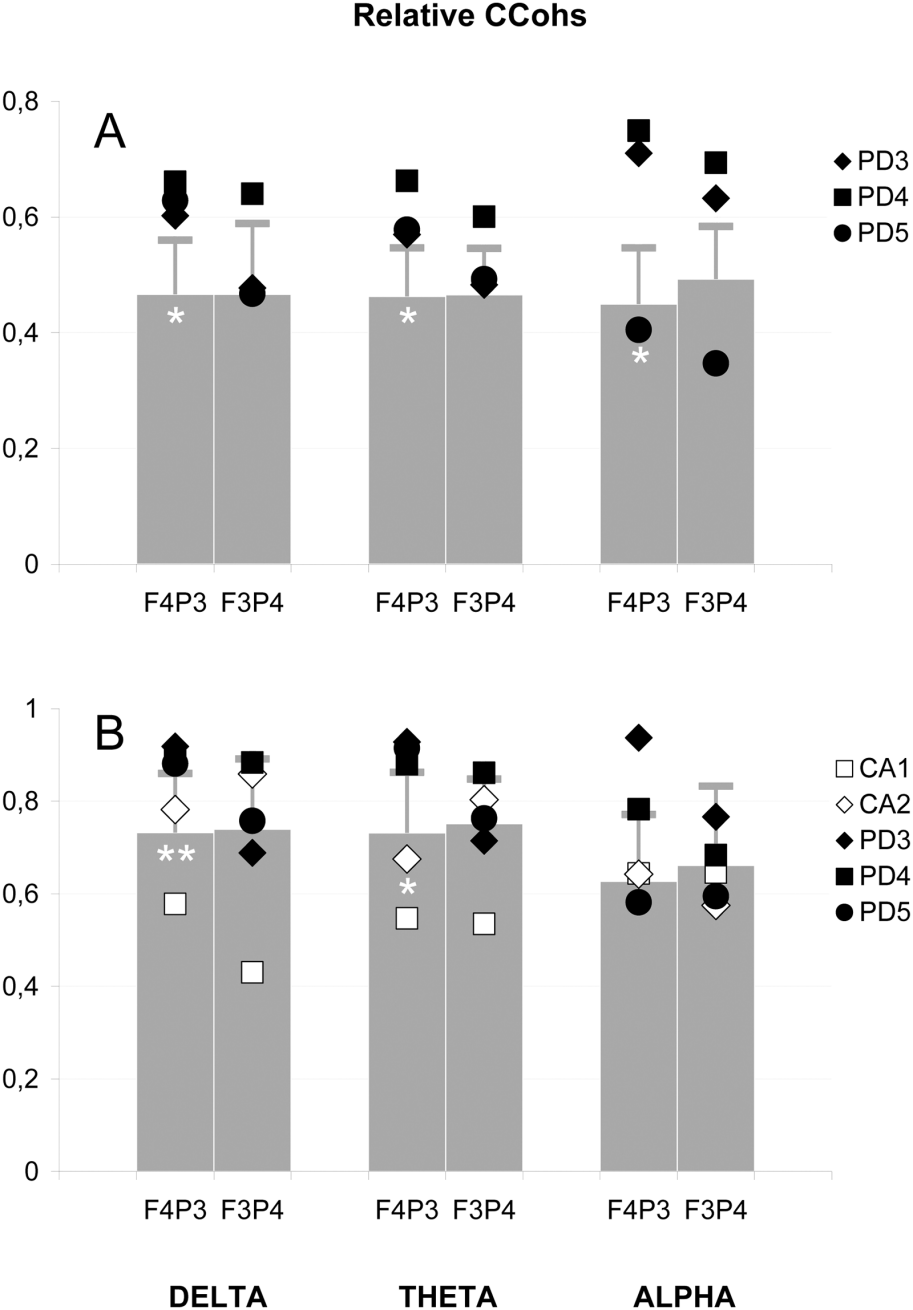
Electrophysiological correlates of the sigmoid bundles: Relative coefficients of coherence (CCohs). (**A**) Relative values averaged for coherent connections of anterior with contralateral posterior leads (see Fig 3A). (**B**) Relative values for separate connection between frontal (F) and contralateral parietal (P) leads of the left (3) and right (4) hemispheres. Bars and horizontal lines represent the mean relative coefficients and standard deviations of control group; black symbols indicate the coefficients of PD patients (see Fig 3) and white symbols show the coefficients of CA patients with callosal agenesis. The number of white asterisks indicates the level of statistical significance of the difference between PD patients and control group: * p<0.05; ** p<0.01.

The *relative* right-anterior-to-left-posterior CCohs averaged in the aforementioned way over five heterologous (crossed) connections and compared among the three groups (Fig 5A) also showed significant prevalence of the PD patients over controls in the delta (0.63 ± 0.03 vs. 0.47 ± 0.09; p = 0.02) and theta (0.60 ± 0.05 vs. 0.46 ± 0.08; p = 0.04) bands. In the alpha band, similarly to the above-mentioned absolute CCohs, such a prevalence was significant for the comparison of the two groups (0.62 ± 0.19 vs. 0.45 ± 0.10; p = 0.03) or for the averaging over the four long right-anterior-to-left-posterior connections, excluding the left central lead (0.57 ± 0.16 vs. 0.38 ± 0.09) which provided significance for this difference (p = 0.03) when the three groups were compared. The mirror relative CCohs did not show significant differences between PD patients and other groups and in each PD patient were lower than the right-anterior-to-left-posterior ones (Fig 5A) (p<0.01 in the theta and alpha bands).

The above-mentioned right-frontopolar-to-left-parietal connection also showed higher *relative* CCohs in the PD patients than in the control group in the delta (0.69 ± 0.11 vs. 0.44 ±0.15; p = 0.04) and theta (0.70 ± 0.13 vs. 0.45 ± 0.14) bands, showing significant prevalence in the alpha band only for the comparison of the two groups (0.64 ± 0.25 vs. 0.42 ± 0.14; p = 0.04). The relative right-frontal-to-left-parietal CCohs were also higher in the AD patients when compared directly with the control group in the delta (0.90 ± 0.02 vs. 0.73 ± 0.13; p = 0.01) and theta (0.91 ± 0.03 vs. 0.73 ± 0.13; p = 0.04) bands, while in the alpha band, such prevalence was significant only for the two PD patients with robust genual remnants (0.86 ± 0.11 vs. 0.63 ± 0.14; p = 0.05) (Fig 5B).

In the CA group, both absolute and relative CCohs topographically corresponding to the SB did not show any significant differences with the other groups.

#### EEG correlates of probst bundles

In all patients, the above-mentioned augmented general prevalence of the number of intrahemispheric HCCohs over that of interhemispheric ones was predominantly due to the long-distance coherence between nontemporal leads. This was most pronounced in the alpha band (Fig 4E), of which the mean absolute CCoh between, for example, parietal and frontal leads was 0.63 ± 0.10, higher than 0.46 ± 0.11 in the control group (p<0.01). The mean intrahemispheric long-distance nontemporal CCohs, calculated for twelve connections in both hemispheres, were significantly higher than in the control group (0.47 ± 0.09 vs. 0.31 ± 0.06; p< 0.001). A similar prevalence of the patients over controls (0.41 ± 0.03 vs. 0.34 ± 0.09) was also observed in the theta band but only in the right hemisphere (p = 0.05) (Fig 3E). For both hemispheres, such a difference in the theta (0.58 ± 0.11 vs. 0.45 ± 0.09; p = 0.02) and delta (0.57 ± 0.11 vs. 0.45 ± 0.09; p = 0.03) bands was detected for the mean CCohs calculated for six long-distance connections of both hemispheres, excluding connections with occipital leads.

The average relative intrahemispheric long-distance CCoh of the patients (0.71 ± 0.10) was higher than in the control group (0.53 ± 0.07; p = 0.001) in the alpha band (Fig 4F). In the theta band, such a prevalence for relative CCohs was significant only in the right hemisphere (0.64 ± 0.07 vs. 0.55 ± 0.08; p = 0.05) and appeared for both hemispheres (0.90 ± 0.15 vs. 0.78 ± 0.10; p = 0.04) when the occipital leads were excluded from the averaging procedure (see above). This procedure also resulted in detecting a similar but left-sided prevalence of the patients in the delta band (0.88 ± 0.13 vs. 0.76 ± 0.09; p = 0.04).

In the delta and theta bands, a significant regional superiority of the long-distance intrahemispheric coherence in the patients predominantly occurred in the centro-frontopolar and parieto-frontopolar connections. These connections together with other CCohs of this type which were significantly higher in the patients were plotted in the illustrative maps of Fig 4E and 4F.

The intrahemispheric short-distance relative CCohs among adjacent nontemporal leads did not show significant differences between patients and controls.

## Discussion

We here provide electrophysiological evidence suggesting a functional anomalous connectivity in CD, which can be related to the aberrant structural connectivity as shown by neuroimaging techniques. EEG coherence patterns were demonstrated to topographically correspond to the white-matter abnormal heterotopic bundles, PB and SB, known as characteristic morphological features of dyscallosal brains [2, 4, 10–12, 19].

EEG coherence has proved sensitive to estimate functional connectivity [20–22, 34], particularly when individual non-specific general levels of coherence are taken into account [23, 24]. This approach confirmed the reduced interhemispheric synchrony between homologous brain regions in absence of callosal connections, as reported previously in the EEG [13–16] and neuroimaging [18] literature. Moreover, this reduction was more widespread (in the anterior-posterior axis) in CA patients (excluding occipital region), while restricted to more posterior regions in PD with a preserved callosal genu. Topographic correspondence between the corpus callosum absence or damage, and decrease in interhemispheric EEG coherence, was also reported by other authors in CD [15], Alzheimer’s disease [35] and partial callosotomy [36]. Interestingly, the atypical PD patient with a very small genu (PD 5) showed decreased anterior CCohs and a spread pattern similar to CA patients. These results suggest that the presence of a robust genu can provide an interhemispheric functional connectivity between the frontal lobes in typical PD.

Interhemispheric coherence decrease was observed in the delta, theta and alpha bands. A similar effect was previously shown for the delta frequencies [13] and other bands [15]. However, the topographic correlation of such decrease with absence of the corpus callosum was not valid for the occipital regions, where the CA and the atypical PD patients did not show reduced CCohs. Some authors reported that the interhemispheric coherence in the occipital cortex is less affected in CD [14, 16] and callosotomy [36], and suggested that the occipital measures might reflect activity of the posterior commissure [14]. This explanation is not likely, however, since the posterior commissure is very small, connecting specifically the mesodiencephalic regions. Nevertheless, a recent report provides strong evidence that it may be related to rewired interhemispheric connections in CD patients [11], which looks promising for further studies. On the other hand, the minimum occipital reduction of the interhemispheric CCohs in the alpha band could also be related to the strong thalamic contribution to the occipital alpha rhythm generation [37]. Beta oscillations are supposedly related to different, spatially restricted thalamocortical sources [38] and generated in the cortex itself [39]. The former hypothesis could partially explain a lack of correlation with callosal absence. Both absence [40] and presence [16] of interhemispheric beta coherence reduction in CD have been reported.

Relative preservation of the general level of interhemispheric coherence in PD patients may also be due to the significantly larger CCohs between anterior areas of one hemisphere and posterior areas of the other, in a configuration consistent with the morphological topography of the SB. It is noteworthy that both anatomical and EEG coherence maps show the same asymmetry with the pronounced prevalence of the right-anterior-to-left-posterior connections over the mirror ones (Figs 1 and 4).

Accordingly, the maps of *long-distance* intrahemispheric CCohs prevalence in patients over controls (Fig 4) are consistent with the morphological topography of the PB and the cingulate bundle as well. Since short-distance longitudinal coherence among adjacent leads in patients did not differ from that of controls, some functional contribution of the PB to long-distance intrahemispheric interactions may be suggested, particularly in the alpha band, which is predominant in the human EEG and normally quite differentiated between frontocentral and posterior regions [37]. Some authors reported no difference between CD patients and controls in intrahemispheric coherence [14]. However, their methodological approach may explain this difference, since they estimated only short-distance connections between adjacent leads, while we here investigated long-distance leads as well.

As to the absence of any correlation between CD and EEG coherence of temporal regions, similar patterns were observed by other authors [15].

In sum, our results demonstrate a topographical correspondence between heterotopic aberrant bundles in CD and functional connectivity between anterior and posterior cortical regions as revealed by EEG coherence analysis. Recent reports have addressed the possible compensatory role of abnormal interhemispheric bundles in the preservation of tactile object recognition in CD [11]. The same, however, could not be inferred by the present data concerning the heterotopic PB and SB, whose functional relation to cognition is still unknown. However, given the high prevalence of abnormal bundles in CD and the fact that most CD patients show a diverse variety of symptoms, from epilepsy to mental retardation [3], it is conceivable that these bundles could be also responsible for some of these clinical presentations. The issue requires further work with a more direct approach of the cognitive functions related to the cortical regions connected by PB and SB.

## Conclusions

The results provide electrophysiological evidence that correlate with the anatomical connectivity of the aberrant bundles in human CD, despite the fact that the nature of this function is still unknown. A higher long-distance intra-hemispheric EEG coherence was found in both hemispheres and was topographically correlated with the aberrant PB. The patients with partial callosal dysgenesis also showed augmented inter-hemispheric heterologous (crossed) EEG coherence with predominance of the right-anterior-to-left-posterior connections over the mirror ones. These asymmetric patterns corresponded to the MRI features of aberrant heterotopic inter-hemispheric SB and might represent the first reported evidence for their functional counterpart.

## References

[1] Richards LJ, Plachez C, Ren T. Mechanisms regulating the development of the corpus callosum and its agenesis in mouse and human. Clin Genet. 2004; 66: 276–289. 15355427 10.1111/j.1399-0004.2004.00354.x

[2] Tovar-Moll F, Moll J, de Oliveira-Souza R, Bramati I, Andreiuolo PA, Lent R. Neuroplasticity in human callosal dysgenesis: a diffusion tensor imaging study. Cereb Cortex 2007; 17: 531–541. 16627861 10.1093/cercor/bhj178

[3] Edwards TJ, Sherr EH, Barkovich AJ, Richards LJ. Clinical, genetic and imaging findings identify new causes for corpus callosum development syndromes. Brain 2014; 137: 1579–1613. 10.1093/brain/awt358 24477430 PMC4032094

[4] Paul LK, Brown WS, Adolphs R, Tyszka JM, Richards LJ, Mukherjee P, et al Agenesis of the corpus callosum: genetic, developmental and functional aspects of connectivity. Nat Rev Neurosci 2007; 8: 287–299. 17375041 10.1038/nrn2107

[5] Siffredi V, Anderson V, Leventer RJ, Spencer-Smith MM. Neuropsychological profile of agenesis of the corpus callosum: a systematic review. Dev Neuropsychol 2013; 38: 36–57. 10.1080/87565641.2012.721421 23311314

[6] Sperry RW, Gazzaniga MS, Bogen JE. Interhemispheric relationships: the neocortical commissures; syndromes of hemisphere disconnection In: Vinken PJ, Bruyn GW, editors. Handbook of Clinical Neurology. Amsterdam: North-Holland Publishing Company, 1969 pp. 273–290.

[7] Lassonde M, Sauerwein H, Chicoine AJ, Geoffroy G. Absence of disconnexion syndrome in callosal agenesis and early callosotomy: brain reorganization or lack of structural specificity during ontogeny? Neuropsychologia 1991; 29: 481–495. 1944857 10.1016/0028-3932(91)90006-t

[8] Ettlinger G, Blakemore CB, Milner AD, Wilson J. Agenesis of the corpus callosum: a behavioural investigation. Brain 1972; 95: 327–346. 4655032 10.1093/brain/95.2.327

[9] Sauerwein HC, Lassonde MC, Cardu B, Geoffroy G. Interhemispheric integration of sensory and motor functions in agenesis of the corpus callosum. Neuropsychologia 1981; 19: 445–454. 7266837 10.1016/0028-3932(81)90074-9

[10] Probst M. Ueber den Blau des balkenlosen Grosshirns, sowie uber Mikrogirie un eterotopie der grauen substanz. Arch Psychiatr Nervenkr 1901; 34: 709–786.

[11] Tovar-Moll F, Monteiro M, Andrade J, Bramati IE, Vianna-Barbosa R, Marins T, et al Structural and functional brain rewiring clarifies preserved interhemispheric transfer in humans born without the corpus callosum. Proc Natl Acad Sci USA 2014; 111: 7843–7848. 10.1073/pnas.1400806111 24821757 PMC4040546

[12] Wahl M, Strominger Z, Jeremy RJ, Barkovich AJ, Wakahiro M, Sherr EH, et al Variability of homotopic and heterotopic callosal connectivity in partial agenesis of the corpus callosum: a 3T diffusion tensor imaging and Q-ball tractography study. AJNR Am J Neuroradiol 2009; 30: 282–289. 10.3174/ajnr.A1361 19001538 PMC7051413

[13] Kuks JB, Vos JE, O'Brien MJ. Coherence patterns of the infant sleep EEG in absence of the corpus callosum. Electroencephalogr Clin Neurophysiol 1987; 66: 8–14. 2431870 10.1016/0013-4694(87)90132-5

[14] Nielsen T, Montplaisir J, Lassonde M. Decreased interhemispheric EEG coherence during sleep in agenesis of the corpus callosum. Eur Neurol 1993; 33: 173–176. 8467828 10.1159/000116928

[15] Koeda T, Knyazeva M, Njiokiktjien C, Jonkman EJ, De Sonneville L, Vildavsky V. The EEG in acallosal children. Coherence values in the resting state: left hemisphere compensatory mechanism? Electroencephalogr Clin Neurophysiol 1995; 95: 397–407. 8536568 10.1016/0013-4694(95)00171-9

[16] Nagase Y, Terasaki O, Okubo Y, Matsuura M, Toru M. Lower interhemispheric coherence in a case of agenesis of the corpus callosum. Clin Electroencephalogr 1994; 25: 36–39. 8174290 10.1177/155005949402500110

[17] Tyszka JM, Kennedy DP, Adolphs R, Paul LK. Intact bilateral resting-state networks in the absence of the corpus callosum. J Neurosci 2011; 31: 15154–15162. 10.1523/JNEUROSCI.1453-11.2011 22016549 PMC3221732

[18] Owen JP, Li YO, Ziv E, Strominger Z, Gold J, Bukhpun P, et al The structural connectome of the human brain in agenesis of the corpus callosum. Neuroimage 2013; 70: 340–355. 10.1016/j.neuroimage.2012.12.031 23268782 PMC4127170

[19] Jakab A, Kasprian G, Schwartz E, Gruber GM, Mitter C, Prayer D, et al Disrupted developmental organization of the structural connectome in fetuses with corpus callosum agenesis. Neuroimage 2015; 111: 277–288. 10.1016/j.neuroimage.2015.02.038 25725467

[20] Livanov MN. Spatial organization of cerebral processes. New York: Wiley; 1977.

[21] Nunez PL, Srinivasan R, Westdorp AF, Wijesinghe RS, Tucker DM, Silberstein RB, et al EEG coherency. I: Statistics, reference electrode, volume conduction, Laplacians, cortical imaging, and interpretation at multiple scales. Electroencephalogr Clin Neurophysiol 1997; 103: 499–515. 9402881 10.1016/s0013-4694(97)00066-7

[22] Coben R, Clarke AR, Hudspeth W, Barry RJ. EEG power and coherence in autistic spectrum disorder. Clin Neurophysiol 2008; 119: 1002–1009. 10.1016/j.clinph.2008.01.013 18331812

[23] Lazarev VV, Pontes A, Mitrofanov AA, deAzevedo LC. Interhemispheric asymmetry in EEG photic driving coherence in childhood autism. Clin Neurophysiol 2010; 121: 145–152. 10.1016/j.clinph.2009.10.010 19951847

[24] Lazarev VV, Pontes A, Mitrofanov AA, deAzevedo LC. Reduced interhemispheric connectivity in childhood autism detected by electroencephalographic photic driving coherence. J Autism Dev Disord 2015; 45: 537–547. 10.1007/s10803-013-1959-8 24097142

[25] Jiang H, van Zijl PC, Kim J, Pearlson GD, Mori S. DtiStudio: resource program for diffusion tensor computation and fiber bundle tracking. Comput Methods Programs Biomed 2006; 81: 106–116. 16413083 10.1016/j.cmpb.2005.08.004

[26] Mori S, Crain BJ, Chacko VP, van Zijl PC. Three-dimensional tracking of axonal projections in the brain by magnetic resonance imaging. Ann Neurol 1999; 45: 265–269. 9989633 10.1002/1531-8249(199902)45:2<265::aid-ana21>3.0.co;2-3

[27] Pajevic S, Pierpaoli C. Color schemes to represent the orientation of anisotropic tissues from diffusion tensor data: Application to white matter fiber tract mapping in the human brain. Magn Reson Med 1999; 42: 526–540. 10467297

[28] Zaveri HP, Williams WJ, Sackellares JC, Beydoun A, Duckrow RB, Spencer SS. Measuring the coherence of intracranial electroencephalograms. Clin Neurophysiol 1999; 110: 1717–1725. 10574287 10.1016/s1388-2457(99)00136-4

[29] Jenkins GM, Watts DG. Spectral analysis and its application. San Francisco: Holden Day; 1968.

[30] Carter GC. Coherence and time delay estimation. Proc IEEE 1987; 75: 236–255.

[31] Wang SY, Liu X, Yianni J, Miall RC, Aziz TZ, Stein JF. Optimizing coherence estimation to assess the functional correlation of tremor-related activity between the subthalamic nucleus and the forearm muscles. J Neurosci Meth 2004; 136: 197–205. 10.1016/j.jneumeth.2004.01.008 15183272

[32] Armitage P, Beery G, Matthews JNS. Statistical methods in medical research. 4th ed Malden: Blackwell Science; 2002.

[33] Nielsen T, Montplaisir J, Lassonde M. Sleep architecture in agenesis of the corpus callosum: laboratory assessment of four cases. J Sleep Res 1992; 1: 197–200. 10607051 10.1111/j.1365-2869.1992.tb00038.x

[34] Tucker DM, Roth DL, Bair TB. Functional connections among cortical regions: topography of EEG coherence. Electroencephalogr Clin Neurophysiol 1986; 63: 242–250. 2419082 10.1016/0013-4694(86)90092-1

[35] Pogarell O, Teipel SJ, Juckel G, Gootjes L, Möller T, Bürger K, et al EEG coherence reflects regional corpus callosum area in Alzheimer’s disease. J Neurol Neurosurg Psychiatry 2005; 76: 109–111. 15608007 10.1136/jnnp.2004.036566 PMC1739328

[36] Montplaisir J, Nielsen T, Côté J, Boivin D, Rouleau I, Lapierre G. Interhemispheric EEG coherence before and after partial callosotomy. Clin Electroencephalogr 1990; 21: 42–47. 2297948 10.1177/155005949002100114

[37] Niedermeyer E, Lopes da Silva FH. Electroencephalography: basic principles, clinical applications, and related fields. 5th ed Philadelphia: Lippincott Williams & Wilkins; 2005.

[38] Papakostopoulos D, Crow HJ, Newton P. Spatiotemporal characteristics of intrinsic and event-related potentials in the human cortex In: Pfurtscheller G, Buser P, Lopes da Silva F, Petsche H, editors. Rhythmic EEG Activities and Cortical Functioning. Amsterdam: Elsevier/Northolland Biomedical Press; 1980 pp179–200.

[39] Lopes da Silva F. EEG: Origin and measurement In: Mulert C, Lemieux L, editors. EEG—fMRI: Physiological Basis, Technique, and Applications. Berlin-Heidelberg: Springer-Verlag, 2010 pp. 19–38.

[40] Hinkley LB, Marco EJ, Findlay AM, Honma S, Jeremy RJ, Strominger Z, et al The role of corpus callosum development in functional connectivity and cognitive processing. PLoS One 2012; 7: e39804 10.1371/journal.pone.0039804 22870191 PMC3411722

